# Cross-Reactive T Cell Immunity to Dengue and Zika Viruses: New Insights Into Vaccine Development

**DOI:** 10.3389/fimmu.2019.01316

**Published:** 2019-06-11

**Authors:** Annie Elong Ngono, Sujan Shresta

**Affiliations:** ^1^Division of Inflammation Biology, La Jolla Institute for Immunology, La Jolla, CA, United States; ^2^Department of Medicine, School of Medicine, University of California San Diego, La Jolla, CA, United States

**Keywords:** cross-reactive T cell response, Dengue, Zika, Epitope Mapping, vaccine development

## Abstract

Dengue virus (DENV) is a member of the Flavivirus family that includes Zika virus (ZIKV), West Nile virus, Japanese encephalitis virus, and yellow fever virus. As the most prevalent of the flaviviruses, DENV is responsible for tens of millions of infections each year. The clinical manifestations of infection with one of the four DENV serotypes (DENV1–4) range from no symptoms to hemorrhagic fever and shock (“severe dengue”), which is fatal in ~25,000 patients annually. Many factors contribute to the development of severe dengue, including the DENV serotype and host expression of certain HLA alleles; however, it now seems clear that pre-existing immunity to DENV—and possibly other flaviviruses—is a major precipitating factor. While primary infection with one DENV serotype elicits strong cellular and humoral immune responses that likely confer long-lived protection against the same serotype, subsequent infection with a different serotype carries an increased risk of developing severe dengue. Thus, primary DENV infection elicits cross-reactive immunity that may be protective or pathogenic, depending on the context of the subsequent infection. Many flaviviruses share high sequence homology, raising the possibility that cross-reactive immunity to one virus may contribute to protection against or pathogenesis of a second virus in a similar manner. In addition, several flaviviruses are now endemic in overlapping geographic regions, underscoring the need to gain more knowledge about the mechanisms underlying cross-reactive immunity to different DENV serotypes and flaviviruses. Here, we review our current understanding of T cell immunity to DENV, focusing on cross-reactivity with other serotypes and flaviviruses such as ZIKV, and the role of DENV-elicited CD4^+^ and CD8^+^ T cells in protection. Recent work in this area supports a beneficial role for cross-reactive T cells and provides new insights into the design of safe and efficient flavivirus/pan-flavivirus vaccines.

## Introduction

Dengue virus (DENV) belongs to the flavivirus genus of the Flaviviridae family, which includes Zika virus (ZIKV), yellow fever virus (YFV), West Nile virus (WNV), and Japanese encephalitis (1). The flaviviruses are transmitted mainly through the bite of *Aedes* genus mosquitoes (namely, *Aedes aegypti* and *Aedes albopictus*), which have expanded to tropical and subtropical areas throughout the globe (2). DENV is currently endemic in more than 128 countries, most of which are developing nations where it imposes major public health and economic burdens. DENV exists as four serotypes (DENV1-4) that share 60–75% amino acid homology (3), and infection with any serotype can be asymptomatic or cause a spectrum of symptoms ranging from mild aches and pains to life-threatening dengue fever/hemorrhagic fever (DF/DHF) leading to shock; this syndrome is now referred to as “severe dengue.” Approximately 100 million new symptomatic cases are reported annually, of which about 2% result in severe dengue, with 25,000 fatalities (4). This number is probably an underestimate considering the challenges in public health surveillance faced by many DENV-endemic countries. In the past few decades, the incidence of DENV has increased and expanded, and half of the global population (~3.6 billion people) is now estimated to be at risk for infection.

Efforts to understand why DENV infection causes such a range of symptoms have been ongoing for more than 60 years. Epidemiological studies showed that severe dengue was more prevalent in children and adults experiencing a secondary infection with a different DENV serotype (heterotypic) compared with the same serotype (homotypic infection) (5, 6). This and other observations suggested that DENV infection may elicit long-term protection against the same serotype but only short-term protection or even enhanced infection with a different serotype. Although multiple factors, such as genetic variation, age, and sex contribute to the development of severe dengue (6), the mechanisms underlying the role played by the immune response in dictating whether DENV infection is protective or pathogenic are the dominant subject of continued research.

The research centers around two mutually non-exclusive hypotheses in which both humoral and cellular immunity to DENV contribute to disease pathogenesis. The first hypothesis stems from the process known as antibody (Ab)-dependent enhancement (ADE), whereby pre-existing cross-reactive Abs enhance the viral burden during subsequent heterotypic infection by promoting Fcγ receptor-mediated cellular uptake. Increasing evidence from humans and mouse models support a direct role for DENV serotype-cross-reactive Abs in severe dengue (6–9). The second hypothesis for the enhanced disease risk during secondary DENV infection is built on studies suggesting a potential role for cross-reactive T cells in DENV pathogenesis (10, 11). According to the original T cell antigenic sin hypothesis, most activated T cells during acute DENV infection are cross-reactive with a previously encountered serotype(s) and have low affinity for the currently infecting serotype, leading to suboptimal control of infection and disease pathogenesis (10). However, at present, the evidence is stronger for a protective rather than a pathogenic role for cross-reactive T cells in DENV infection (8). The relative contribution of host humoral *vs*. cellular immunity to the control of DENV infection is of great relevance for the development of safe and effective DENV vaccines. The only currently licensed DENV vaccine, Dengvaxia® (CYD-TDV, Sanofi Pasteur), expresses the DENV E from YFV vector backbone which include the YF NS proteins and elicits Ab responses to DENV E protein (the major target of anti-flavivirus Ab responses) but not T cell responses to DENV NS proteins (the major targets of anti-DENV T cell response, as discussed below). Multiple clinical trials have revealed that Dengvaxia® elicits suboptimal Ab responses against all four DENV serotypes, and the vaccine-induced Ab responses wane within 3–4 years after vaccination (12, 13). In particular, analysis of pooled data from two phase 3 clinical trials of Dengvaxia® (CYD14 and CYD15) of Asian-Pacific and Latin American cohorts, respectively, revealed that young children (2–5 years of age) who had not previously been exposed to DENV were at increased risk for developing severe dengue upon vaccination (13, 14). This was ascribed to unequal Ab-mediated protection and low T cell responses elicited by the vaccine against the four DENV serotypes (15, 16), which may set the scene for ADE during subsequent infections. Thus, these Dengvaxia® findings support our hypothesis that the beneficial components of T cell responses should be harnessed in the design of optimal DENV vaccines (8).

A second flavivirus that has risen to the forefront of basic and clinical research in recent years is ZIKV. This virus was discovered in 1947 in Uganda, but the more recent outbreaks have brought worldwide attention to its potentially devastating effects, which include congenital ZIKV syndrome in infants born to ZIKV-infected mothers, and neurological disorders such as Guillain-Barre syndrome in adults (17). DENV and ZIKV share approximately 44% sequence identity at the amino acid level, are both widely distributed throughout the globe, and have overlapping areas of endemicity. These aspects, together with the studies of immunity to heterotypic DENV serotypes, raised the possibility that primary infection with DENV or ZIKV may induce an immune response cross-reactive with the reciprocal virus. Current work suggests that the mechanisms underlying cross-reactive humoral and cellular immunity resulting from ZIKV or DENV primary infection are as complex as those underlying heterotypic DENV infections (8). Here, we review recent developments in our understanding of the protective vs. pathogenic roles of DENV-elicited T cells in the context of subsequent infections with other DENV serotypes or ZIKV. The evidence presented here provides new insights into the need to consider both Ab and T cell immunity in the development of safe and effective flavivirus vaccines.

## Immunodominance of DENV Serotype-Specific T Cell Responses in Humans and Animal Models

DENV is a positive-sense single-stranded RNA virus with an ~11 Kb genome. As is the case for all flaviviruses, the DENV genome encodes three structural proteins (capsid [C], precursor membrane [prM], envelope [E]) and seven non-structural proteins (NS1, NS2A, NS2B, NS3, NS4A, NS4B, NS5). A comprehensive understanding of the immunodominant T cell epitopes in each flaviviral proteome, the parameters that influence their immunodominance, and the extent to which each epitope elicits T cell cross-reactivity to heterotypic serotypes/viruses, are of crucial importance for vaccine design.

Substantial effort using patient samples and mouse models has resulted in the identification of many CD4^+^ and CD8^+^ T cell immunodominant epitopes during DENV and ZIKV primary and secondary infections. Some studies have taken the approach of testing isolated T cell responses to overlapping peptide libraries encompassing a viral protein or the entire viral proteome. Others have used predictive computational algorithms to identify the peptides most likely to interact with a given set of HLA or MHC molecules, which narrows the spectrum of epitopes to be screened. Studies using overlapping peptides spanning the DENV2 proteome identified NS3 as the most frequent target of the T cell response in DENV-infected patients in Thailand and India (18, 19). Using a similar approach with T cells isolated from DENV-infected Singaporean adults, Rivino et al. showed that CD8^+^ T cells targeted mostly NS3 and NS5 proteins, whereas the CD4^+^ T cell response was directed largely against C, E, and NS1 proteins (20). In contrast to the overlapping peptide approach, Weiskopf et al. used a T cell epitope prediction program to identify over 200 new CD8^+^ T cell epitopes in DENV using peripheral blood mononuclear cells (PBMC) obtained from almost 200 healthy blood donors from Sri Lanka, and their results revealed that NS3, NS4B, and NS5 are the major CD8^+^ T cell targets (21). Using a similar comprehensive approach of identifying immunogenic epitopes with broad coverage of HLA types expressed worldwide, Weiskopf and colleagues demonstrated that NS3, NS5, and the structural protein C are the major target proteins of the anti-DENV CD4^+^ T cell response in humans (22–24). Collectively, these studies indicate that NS3 and NS5 are the major targets of the anti-DENV CD4^+^ and CD8^+^ T cell responses in humans.

**Figure 1:**
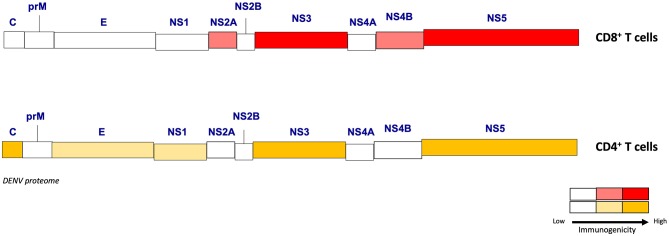
Immunogenicity of the DENV proteome for CD4^+^ and CD8^+^ T cells. Different levels of immunogenicity (from low to high) are represented for CD8^+^ and CD4^+^ T cells among structural (C, M, E) and non-structural (NS1, 2A, 2B, 3, 4A, 4B, 5) DENV proteins. This schematic highlights the DENV proteins that are able to induce strong T cell responses.

However, a challenge of examining T cell immunity in humans is that an accurate infection history is not always available. Non-Human Primates (NHP) do not develop severe dengue disease manifestations, including vascular leakage, and need high maintenance and cost for each study. The majority of mouse models are genetically modified to allow effective replication and development of clinical outcomes. Although, many of these mouse models lack one or more components of the IFN system, as DENV cannot inhibit the IFN signaling in mouse cells (unlike in human cells) to establish robust replication, they are still important as the first step to study protective/pathogenesis effects and test vaccine or antiviral drug candidates. In the context of investigating T cell responses, the antigenic load dictates the level of T cell responses to viruses (25), and thus WT mice, which do not support robust DENV replication, are not ideal for investigating the contribution of T cells in modulating viral replication and disease manifestations. Despite their limitations, these immunodeficient mouse models have therefore proven to be invaluable in identifying virus-specific and cross-reactive T cell epitopes. In particular, type I interferon (IFN) receptor (Ifnar1)-deficient mice, which are more susceptible than wild-type strains to DENV infection (26), backcrossed to HLA transgenic mice, which enable investigation of epitopes likely to be immunodominant in humans (27), have provided key insights into immunodominance patterns during primary and secondary DENV infections. Using a model of primary DENV2 infection in HLA-A^*^0201, A^*^0101, A^*^1101, B^*^0702, and DRB1^*^0101 transgenic *Ifnar1*^−/−^ mice, we first reported that the anti-DENV2 CD8^+^ T cells recognize predominantly NS3 and NS5 epitopes, and CD4^+^ T cells recognize C, NS3, and NS5 epitopes (27). Moreover, the mice expressing known protective HLA molecules, such as HLA-B^*^0702, elicited broader and higher magnitude responses than an HLA associated with susceptibility to DENV infection (HLA-A^*^0101), in agreement with the human data (21). In contrast with DENV2, our model of primary DENV3 infection in HLA transgenic *Ifnar1*^−/−^ mice revealed that DENV3 induced a T cell response directed mainly (33%) against C, prM, and E epitopes, whereas only 3% of the T cell response in DENV2-infected mice was against these proteins (28). Similar results were observed in humans, where the CD8^+^ T cell response in DENV3-infected individuals was directed more toward the structural proteins than the nonstructural proteins (29). We next modeled secondary homotypic and heterotypic DENV infections in HLA transgenic *Ifnar1*^−/−^ mice and observed that CD8^+^ T cell responses were broad (targeting both structural and NS proteins) following primary and homotypic secondary infection, whereas CD8^+^ T cell responses following heterotypic secondary infection focused toward the conserved NS proteins (28), as observed in humans with natural DENV reinfections (19, 21, 30). Collectively, the HLA transgenic mouse model and human data indicate that (i) the CD8^+^ T cell responses are mostly directed against nonstructural proteins NS3, NS4B, and NS5 for DENV1, 2, and 4 and both structural proteins (C, M, and E) and nonstructural proteins (NS3, NS4B, and NS5) for DENV3, (ii) CD4^+^ T cells preferentially recognize epitopes in C, NS3 and NS5 proteins, and (iii) primary infection is dominated by serotype-specific T cells, whereas memory T cells that recognize conserved epitopes are expanded together with naïve serotype-specific T cells during secondary infection (Figure 1). Thus, while epitopes from all DENV proteins can potentially be recognized by CD4^+^ and CD8^+^ T cells, the precise pattern of immunodominance depends on the T cell type, the infecting serotype, and the individual's history of infection (Table 1).

**Table 1:** Parameters that influence the immunodominance of the T cell responses to DENV and ZIKV.

**Parameters**	**References**	**Conclusions**
Cell types	(27, 30)	Distinct immunodominance patterns between CD4^+^ and CD8^+^ T cells
Serotypes	(23, 28)	DENV3 showed a distinct immunodominance compared to DENV1,2, 4 serotypes
Sequence of infection	(28)	The immunodominance patterns of T cells are shaped by the serotype infecting during the primary vs. secondary infection
Primary *vs*. secondary infections	(21, 28, 31, 32)	During primary infection, serotype-specific epitopes are targeted by CD4^+^ and CD8^+^ T cells, although conserved epitopes are mostly targeted during secondary infections
HLA alleles	(21, 23, 27, 33–35)	Magnitude, frequency, and breadth of T cell responses are associated with particular HLA alleles (protective or susceptible HLA restriction)
Pre-existing immunity to DENV	(36–39)	DENV-immunity modulated ZIKV immunodominance patterns

## Immunodominance of DENV-Elicited ZIKV Cross-Reactive T Cell Responses

Recent evidence indicate that DENV-elicited T cells are cross-reactive also with ZIKV. Our study using a sequential model of DENV2 followed by ZIKV infection in *Ifnar1*^−/−^ HLA transgenic B^*^0702 and A^*^0101 mice showed that DENV2-elicited CD8^+^ T cells recognized epitopes in ZIKV NS proteins, of which ~40% were located in NS3 and ~20% each in NS2A, NS4B, and NS5 (37). We also defined DENV2-elicited ZIKV cross-reactive epitopes in WT and congenic *Ifnar1*^−/−^ mice in the C57BL/6 genetic background (38). We observed that DENV2-elicited CD8^+^ T cells mainly recognized epitopes in ZIKV NS3, NS5, prM and E, and that these cross-reactive CD8 T cell responses dominated during ZIKV infection in *Ifnar1*^−/−^and WT C57Bl/6 mice with Ifnar1 blockade (via pre-treatment with blocking anti-Ifnar1 Ab). These mouse data have been validated by a human study that compared ZIKV cross-reactive T cells from naïve *vs*. DENV-exposed individuals from two DENV-endemic countries (Sri Lanka and Nicaragua) (36). Specifically, Grifoni and colleagues observed not only that DENV-specific CD8^+^ T cells recognized ZIKV epitopes but also that prior exposure to DENV altered the pattern of epitope immunodominance (36). Thus, *in vitro* screening of CD8^+^ T cells from DENV-immune individuals showed a dominant response to epitopes in ZIKV non-structural proteins (mainly NS3 and NS5), whereas cells from DENV-naive individuals targeted C, E, and prM. In line with this finding, a study with West African patients exposed to ZIKV and/or DENV showed that T cell cross-reactivity was more strongly directed against epitopes from the DENV and ZIKV NS3 helicase region (71% sequence homology) than the protease region (53% sequence homology) (40). Similarly, in another study of DENV-immune individuals, several epitopes in ZIKV NS3 were recognized by cross-reactive DENV-elicited CD4^+^ and CD8^+^ T cells, whereas fewer cross-reactive epitopes were located in ZIKV C protein (41). The high level of sequence conservation among flaviviral NS3 proteins most likely explains the immunodominant response to NS3. Collectively, these mouse and human studies have demonstrated that DENV-elicited CD8^+^ and CD4^+^ T cells are highly cross-reactive with ZIKV. Additionally, in the context of reciprocal infection, mouse studies have already shown that ZIKV-elicited CD8^+^ T cells are cross-reactive with DENV. Further studies with animal models and humans in particular are now necessary to define the precise features of the cross-reactive ZIKV-elicited T cells against DENV and vice-versa.

## Pathogenic vs. Protective Functions of DENV-Elicited Cross-Reactive T Cells

Earlier studies with DENV-infected humans suggested that T cells may be playing a pathogenic role during secondary infection with heterotypic DENV. In particular, Green et al. reported that activated T cells (CD69^+^) were more abundant in patients with severe dengue compared with mild disease or no symptoms (42). In addition, Mongkolsapaya et al. observed a higher frequency of DENV-reactive CD8^+^ T cells with low affinity in patients experiencing severe dengue compared with mild disease (10). These results were in agreement with other studies demonstrating different immune profiles (cytokine production and cytotoxicity) for CD8^+^ and CD4^+^ T cells from severe dengue patients compared with mild dengue patients (19, 43). For instance, NS3-specific CD8^+^ T cells from donors with severe dengue had a higher production of tumor necrosis factor (TNF) vs. IFNγ compared with children with mild dengue (19). Along the same line, CD4^+^ T cells from Thai school children with secondary DENV infection produced more TNF when stimulated with heterotypic DENV antigens compared with homotypic antigens *in vitro* (43). In support of these human studies implicating a pathogenic role for cross-reactive T cells during DENV infections, a study with wildtype C57BL/6 mice demonstrated that adoptive transfer of DENV1-elicited CD8^+^ T cells into naïve mice triggered some signs of disease following DENV2 challenge (44). However, wildtype C57BL/6 mice are highly resistant to DENV infection, do not develop vascular leakage, a hallmark of severe dengue, and the T cell response in wildtype mice may be limited due to a small antigenic load (8). Thus, at present, direct evidence linking cross-reactive T cells to severe dengue pathogenesis is lacking.

On the other side of the protective *vs*. pathogenic immunity debate, increasing evidence supports a protective role for DENV serotype-cross-reactive CD4^+^ and CD8^+^ T cells. In particular, comprehensive epitope identification studies have revealed that CD4^+^ and CD8^+^ T cell responses restricted to HLA molecules associated with a “low risk” of severe dengue display more robust and polyfunctional responses than cells restricted to “high-susceptibility” HLA molecules (21, 23). Weiskopf et al. used healthy blood donors from a DENV hyperendemic country, Sri Lanka, with 80% of the general population being DENV seropositive. Detailed analysis of DENV epitope-specific CD8^+^ T cell responses in healthy DENV-immune individuals showed no differences in the magnitude, phenotype, functionality, and avidity of the responses to serotype-specific and conserved epitopes (21). Notably, the magnitude, frequency, and breadth of the memory T cell responses were dictated by the restricting HLA, in that presentation by protective HLA molecules (B^*^3501, B^*^0702, and B^*^5801) elicited strong and broad responses compared with susceptible molecules (HLA-A^*^2402 and A^*^0101) (21). Consistent with these findings, examination of the transcriptional signatures of PBMC from asymptomatic and symptomatic DENV-infected individuals revealed higher expression of genes related to adaptive immunity and T cell activation in the asymptomatic subjects compared with the patients with severe dengue (45). More recently, a higher frequency of DENV-specific T cells was detected in patients with mild dengue relative to those with severe dengue (46). Collectively, these human data implicate a protective role of T cells in dengue disease control. However, more studies evaluating the precise phenotype and epitope specificity of CD4^+^ and CD8^+^ T cells during various phases of DENV infection (acute and convalescence) are needed to better understand the contribution of T cells in protective vs. pathogenic immunity.

In line with the human data, studies using models of DENV infection in gene-deficient mice lacking type I IFN receptor or both type I and II IFN receptors, which can support robust DENV replication and manifest severe dengue-like disease, have provided direct evidence in support of a protective role for serotype-cross-reactive T cells (47–49). Higher mortality and viral tissue burden were observed in mice that had been primed with DENV4, depleted of CD8^+^ T cells, and then challenged with DENV2 compared with similarly treated CD8^+^ T cell-sufficient mice (48). Transfer of DENV4-elicited CD8^+^ T cells to naive mice reduced the viral burden upon challenge with DENV2, whereas CD8^+^ T cells were not required for protection after challenge with homotypic DENV4 (48). Moreover, DENV2-cross-reactive CD8^+^ T cells contributed to the control of viral burden in HLA-B^*^07 transgenic mice after vaccination with variant peptides from other DENV serotypes (49). Taken together, the human and mouse data indicate that serotype-cross-reactive CD8^+^ T cells are important mediators of protection against DENV infection.

Recent human studies have also begun to identify potential T cell-based correlates of protection against DENV in the context of both natural infections (21, 50, 51) and vaccination (TV003/TV005) (24, 50). A protective CD8^+^ T cell response appears to be driven by DENV-specific effector memory T_EM_ (CCR7^−^ CD45RA^−^) and T_EM_ expressing CD45RA, known as T_EMRA_ (CCR7^−^ CD45RA^+^). Both CD8^+^ T_EM_ and T_EMRA_ subsets produce IFNγ and TNF, express cytotoxic molecules such as granzyme B or CD107a, and upregulate PD1. A positive correlation between the expression of PD-1 and the magnitude of the CD8^+^ T cell response restricted by HLA^*^B3501, associated with a low risk of developing severe dengue, has been observed (51). CD8^+^ T_EM_/T_EMRA_ cells in blood from dengue-infected adults during acute phase (52) expressed high levels of CLA (cutaneous lymphocyte-associated antigen), a skin homing receptor, and chemokine receptors CXCR3, CCR5, and CXCR6, which support T cell migration to inflamed tissues (52). In this study, CLA-expressing DENV-specific CD8^+^ and CD4^+^ T cells were able to home to the skin during acute DENV infection, suggesting that these cells may be retained in the skin as first line of defense during DENV reinfection. Taken together, these observations indicate that the phenotypes of DENV-specific CD8^+^ T cells that are associated with protection include T_EM_ and T_EMRA_ subsets expressing IFNγ, TNF, cytotoxicity markers, PD1, CLA, and chemokine receptors CXCR3, CCR5, and CXCR6, and they suggest that the anti-DENV CD8^+^ T cells may exert antiviral effects both through production of cytokines such as IFNγ and TNF and cytotoxicity.

The phenotype of DENV-specific CD4^+^ T cells that are associated with protection have also begun to be investigated. In a study involving healthy donors, a higher frequency of DENV-specific CD4^+^ T_EMRA_ that produce more IFNγ than CD4^+^ T_EM_ or T_CM_ cells and express CXCR3 and cytolytic molecules (e.g., granzyme B and perforin) was found in the blood of individuals classified as primary or secondary infections compared with dengue-negative healthy individuals (53). There was no significant difference between primary vs. secondary infection groups. In this study, secondary infection referred to seropositive healthy donors with neutralizing titers to more than one serotype and primary infection corresponds to donors with neutralizing titers to only one serotype. These IFNγ-producing cytotoxic CD4^+^ T cells (CTL) were more abundant in donors expressing HLA associated with protection (HLA DRB1^*^0401) than those associated with susceptibility to DENV infection (HLA DRB1^*^0802) (53). Recently, single-cell RNA sequencing identified DENV-specific CD4^+^ T CTL as a distinct population (54). In addition to CD4^+^ CTLS, CD4^+^ follicular T helper cells (Tfh) that express CXCR5^+^ have been observed in PBMCs from convalescent DENV-infected patients after stimulation with DENV peptides (30). A recent study of DENV-infected children in Thailand showed that peripheral Tfh cells were expanded and activated (PD-1^hi^, CD38^+^) during acute infection, and a higher frequency of activated Tfh cells were observed in patients with severe disease relative to those with mild disease (55). Thus, the magnitude and precise phenotype of CD4^+^ T cells that are polarized to cytotoxic Th1 and Tfh cells may serve as important correlates of protection against DENV infections.

Since the recent emergence of ZIKV in the Americas, studies have begun to examine how prior infection with DENV influences T cell immunity and the pathogenesis of ZIKV. Similar to the observations in patients with heterotypic DENV secondary infection, CD4^+^ and CD8^+^ T cells from DENV-immune humans (naturally infected or vaccinated) are mostly T_EM_/T_EMRA_, express more polyfunctional cytokines, and display higher levels of cytotoxicity and activation markers (granzyme B, PD-1) than cells from ZIKV-infected DENV-naïve individuals (36, 40, 56). Importantly, studies modeling sequential DENV followed by ZIKV infection in a variety of mouse strains, including *Ifnar1*^−/−^ HLA transgenic, wildtype, and *Ifnar1*^−/−^, have demonstrated that DENV-elicited CD8^+^ T cells are required for protection against ZIKV infection in both non-pregnant and pregnant mice (37–39, 57). Thus, these mouse model studies suggest that prior DENV immunity can afford cross-protection against ZIKV via CD8^+^ T cells.

Altogether, evidence to date supports an important role for DENV-elicited CD8^+^ T cells in mediating protection against heterotypic DENV and ZIKV infections. In comparison with CD8^+^ T cells, the function of cross-reactive CD4^+^ T cells during sequential infection with heterotypic DENV serotypes or with DENV and ZIKV has been explored to a lesser extent. Mouse models are thus poised to reveal the precise roles of the cross-reactive CD4^+^ T cell responses and the interplay between the pre-existing DENV-elicited CD8^+^ and CD4^+^ T cell responses during various reinfection settings.

## Designing Anti-Flaviviral Vaccines to Induce Both T Cell and Humoral Immunity

The quest for a safe and effective DENV vaccine has been ongoing for nearly 70 years. However, one of the most successful human vaccines designed to date is the live-attenuated yellow fever virus vaccine (YFV-17D), which has significantly reduced the incidence of this important flaviviral disease worldwide. YFV-17D induces strong Ab and T cell responses that mediate long-term protection in humans (58), and the YFV-17D-elicited CD8^+^ T cell response includes terminally differentiated CD8^+^ T_EMRA_ cells that express PD-1 and are highly proliferative and polyfunctional (59). To produce Dengvaxia®, Sanofi Pasteur generated chimeric viruses with prM and E proteins from DENV1-4 and NS proteins from YFV-17D. This is currently the only licensed DENV vaccine and it has several drawbacks. The vaccine elicits suboptimal Ab responses with differential neutralizing activity against the four DENV serotypes; for example, titers of anti-DENV2 neutralizing Ab are particularly low (15), which could explain the elevated risk of severe dengue observed in vaccinated 2- to 5-year-olds (14). In addition, Dengvaxia® failed to protect naïve individuals (14, 60). Based on these outcomes, Dengvaxia® is recommended only for DENV-immune individuals older than 9 years of age (13). The failure of Dengvaxia® to fully protect may well be due to the absence of DENV NS proteins, which, as noted here, are the predominant targets of DENV-specific and cross-reactive T cell responses. In contrast, the vaccine's beneficial effect in DENV-immune individuals could result from a combination of the Ab response and stimulation of pre-existing DENV-elicited, YFV NS3-cross-reactive T cells (61). This possibility is supported by studies showing that Dengvaxia® induced a strong anti-YFV NS3 CD8^+^ T cell response in DENV-naïve individuals but a weak anti-DENV CD8^+^ T cell response in DENV-immune individuals (16, 62). This observation substantiates the need to include immunodominant DENV-specific proteins in vaccines against DENV.

Two different DENV vaccine candidates (from Takeda and NIH/Butantan) that also represent live-attenuated chimeric viruses are now in phase III trials and have shown promising results in eliciting both cellular and humoral immune responses. TV003 is a tetravalent formulation composed of DENV1–3 and a chimeric DENV2 that includes prM and E from DENV2 and other proteins from DENV4. A single dose of TV003 has been shown to induce neutralizing Abs in up to 90% of vaccinees (63) and polyfunctional T cell responses that mainly target the most conserved epitopes in NS3 and NS5 (50). Another live-attenuated DENV vaccine candidate, TAK-003 from Takeda, is a tetravalent formulation of DENV2 and prM and E from DENV1, DENV3, and DENV3 within the same DENV2 backbone. This vaccine candidate induces a polyfunctional CD8^+^ T cell response against DENV2 NS1, NS3, and NS5 proteins that is cross-reactive on the same proteins from DENV1, 3, and 4 (64). However, the absence of nonstructural proteins from DENV1, 3, or 4 in this vaccine may be limiting, given that the pattern of immunodominant epitopes is influenced not only by the serotype but also by the vaccinee's infection history (28). In addition, it is important to note that the cross-reactive T cell response to heterologous flaviviral infection confers only short-term protection (39). Long-term monitoring of the vaccinated individuals is necessary to determine whether vaccines can induce a sustained cross-reactive T cell response, which is the ideal outcome.

**Figure 2:**
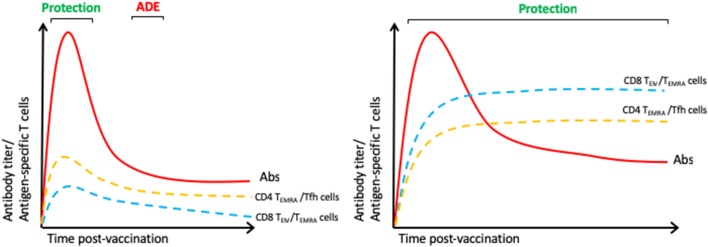
Immune responses of an ideal DENV/ZIKV vaccine. We hypothesize that the licensed DENV vaccine elicits suboptimal antibody responses and inefficient CD4^+^ and CD8^+^ T cell responses **(Left)** and that an ideal vaccine should induce balanced Ab, CD8^+^ (T_EM_/T_EMRA_) and CD4^+^ (Th1, CTL, and Tfh) T cell responses **(Right)**.

Several ZIKV vaccine candidates have been tested in animal models (65). Of these, prM/E DNA vaccines have shown the most promising results, but they appear to elicit poor T cell responses, despite high neutralizing anti-E protein Ab titers, in both mice (66) and rhesus monkeys (67). Moreover, the monkey study demonstrated protection against ZIKV infection that correlated with the Ab titer, but transfer of Abs from vaccinated to naïve monkeys conferred only partial protection against subsequent ZIKV infection (67), suggesting that Ab responses alone may be insufficient for full protection. Importantly, ZIKV-elicited Abs are able to induce ADE of DENV infection *in vitro* and *in vivo* (68–70). This finding underscores the potential dangers of vaccinating DENV-immune individuals with a ZIKV vaccine that induces only an Ab response. In contrast, DENV-elicited cross-reactive CD8^+^ T cells were able to protect against ZIKV in virgin and pregnant mice (37–39). There are direct evidences showing that DENV or ZIKV protein/epitopes induced protection in mice via T cells. Costa et al. showed that Balb/c mice vaccinated with DNA vaccines based on full-length or helicase domain NS3 of DENV2 are protected against lethal challenge (71). Similarly, we showed protection mediated by CD8^+^ T cells in AG129 and wild-type mice vaccinated with VRP expressing the DENV2-E protein ectodomain (DENV2 E85-VRP) (72). In this study, CD8^+^ T cells from vaccinated mice significantly contribute to the reduction of viral RNA in tissues (72). Vaccination of human relevant peptides HLA-restricted, identified as immunodominant epitopes, contribute to the reduction of DENV/ZIKV viral burden (37, 49). We demonstrated that peptide immunization of HLAB^*^0702 and HLAA^*^0101-restricted epitopes contribute to protection. Based on studies implicating an important role for cross-reactive T cells in mediating protection against DENV and ZIKV, a DENV/ZIKV vaccine could be designed to induce balanced Ab, CD4^+^ Tfh, CD4^+^ Th1, and CD8^+^ T cell responses in order to confer long-lived protection that is mediated by both humoral and cellular immunity (Figure 2).

In conclusion, the studies reviewed here strongly support a key role for T cells in protecting against DENV and ZIKV infections. Comprehensive studies that examine the T cell responses in humans at several time points during acute DENV/ZIKV infections and include patients exhibiting a wide spectrum of clinical disease manifestations (including no symptoms), infection history, ethnicity, and geographic location now need to be conducted. Knowledge gained from these studies will provide insights into the design of safe and effective DENV and ZIKV vaccines that elicit balanced Ab and T cell responses. Several studies have already shown that DENV/ZIKV proteomes contain cross-reactive immunogenic epitopes that can elicit polyfunctional effector T cell responses. Thus, we propose that pan-flavivirus vaccine candidates that take into consideration of such epitopes should be designed to solve the global problem associated with genetic and antigenic similarity and co-circulation of DENV and ZIKV.

## Author Contributions

AEN wrote the review, and SS edited the review drafts.

### Conflict of Interest Statement

The authors declare that the research was conducted in the absence of any commercial or financial relationships that could be construed as a potential conflict of interest.
